# A clinical and molecular pathology prediction model for central lymph node metastasis in cN0 papillary thyroid microcarcinoma

**DOI:** 10.3389/fendo.2023.1075598

**Published:** 2023-02-02

**Authors:** Teng Ma, Lulu Wang, Xueyan Zhang, Yafei Shi

**Affiliations:** ^1^ Department of Thyroid Surgery, Affiliated Hospital of Jining Medical University, Jining, Shandong, China; ^2^ Qingdao Medical College, Qingdao University, Qingdao, Shandong, China; ^3^ Department of Cardiovascular Surgery, Affiliated Hospital of Qingdao University, Qingdao, Shandong, China

**Keywords:** papillary thyroid microcarcinoma, central lymph node metastasis, molecular pathology markers, nomogram, prediction model

## Abstract

**Background:**

The frequency of thyroid cancer has rapidly increased in recent years globally. Thus, more papillary thyroid microcarcinoma (PTMC) patients are being diagnosed, including clinical lymph node-negative (cN0) patients. Our study attempted to develop a prediction model for assessing the probability of central lymph node metastasis (CLNM) in cN0 PTMC patients.

**Methods:**

A total of 595 patients from the Affiliated Hospital of Qingdao University (training cohort: 456 patients) and the Affiliated Hospital of Jining Medical University (verification cohort: 139 patients) who underwent thyroid surgery between January 2020 and May 2022 were enrolled in this study. Their clinical and molecular pathology data were analyzed with multivariate logistic regression to identify independent factors, and then we established a prediction model to assess the risk of CLNM in cN0 PTMC patients.

**Results:**

Multivariate logistic regression analysis revealed that sex, Hashimoto’s thyroiditis (HT), tumor size, extrathyroidal extension, TERT promoter mutations and NRAS mutation were independent factors of CLNM. The prediction model demonstrated good discrimination ability (C-index: 0.757 and 0.753 in the derivation and validation cohorts, respectively). The calibration curve of the model was near the optimum diagonal line, and decision curve analysis (DCA) showed a noticeably better benefit.

**Conclusion:**

CLNM in cN0 PTMC patients is associated with male sex, tumor size, extrathyroidal extension, HT, TERT promoter mutations and NRAS mutation. The prediction model exhibits good discrimination, calibration and clinical usefulness. This model will help to assess CLNM risk and make clinical decisions in cN0 PTMC patients.

## Introduction

The most prevalent endocrine malignancy is thyroid carcinoma, and papillary thyroid carcinoma (PTC) accounts for nearly 90%, including probably 50% microcarcinoma (PTMC) (1, 2). PTC with a maximal diameter of less than 1 cm is referred to PTMC. In the past, most PTMCs were diagnosed after the excision of benign thyroid tumors, but now with the popularity of health checkups and widespread use of ultrasound-guided fine needle aspiration biopsy (US-FNAB), the diagnosis rate of PTMC is on the rise (3).

Papillary thyroid carcinoma itself has relatively indolent biological characteristics and superadd the small tumor burden; PTMC patients usually have a good prognosis after treatment (4). The 2022 National Comprehensive Cancer Network (NCCN) advises against utilizing prophylactic CLND to treat T1 and T2 cN0 PTC (5). Nevertheless, the sensitivity of preoperative detection, such as ultrasound, is low for assessing the central lymph node (CLN) state (6); thus, the high probability of occult CLNM cannot be ignored even in cN0 PTMCs (7).

Numerous studies have analyzed the risk factors for CLNM in cN0 PTMC. Our study aimed to create a validated predictive model including preoperatively determinable clinical data and molecular markers, hoping to provide valuable information for clinicians about facilitating the individualized prediction of CLNM in cN0 PTMC patients and guide precise treatment.

## Materials and methods

### Study design and population

Our study enrolled 2 independent sets of patients from 2 independent medical centers. A total of 456 patients diagnosed with cN0 PTMC in the Thyroid Surgery Department of the Affiliated Hospital of Qingdao University from January 2020 to May 2022 were included as the training cohort. A total of 139 patients were diagnosed with cN0 PTMC in the Thyroid Surgery Department of the Affiliated Hospital of Jining Medical University at the same time as the validation cohort. All operations were performed by surgeons who completed more than 400 surgeries per year. This study was approved by the Affiliated Hospital of Jining Medical University ethics committee (2022C103). Consent has been obtained from each patient or subject after a full explanation of the purpose and nature of all procedures used.

The inclusion criteria were as follows: (1) patients were diagnosed with cN0 PTMC through preoperative ultrasonography (US), computed tomography (CT) and US-FNAB; (2) patients underwent total thyroidectomy with CLND, unilateral thyroid lobectomy and isthmectomy with CLND; and (3) patients underwent BRAF V600E, TERT, RET, HRAS, KRAS, and NRAS genetic testing.

The exclusion criteria were as follows: (1) patients diagnosed with recurrent thyroid tumors (only primary thyroid carcinoma patients were included); (2) patients who had previously undergone neck radiation therapy or other types of surgery; and (3) patients with incomplete medical records.

### Data collection

Data collected included sex, age, surgical method, thyroid peroxidase antibody (TPOAb), thyroglobulin antibody (TGAb), thyroid hormone receptor antibody (TRAb), microcalcification foci, positive lymph node number, BRAF gene mutation(V600E), TERT promoter mutation(C228, C250), RET/PTC chromosomal rearrangement(RET/PTC1, RET/PTC3), HRAS gene mutation(Q61), KRAS gene mutation(G12, G13), and NRAS gene mutation(Q61). All data from preoperative examinations and postoperative pathological reports, such as cervical ultrasonic and CT, laboratory examination, and gene test results, were evaluated.

All preoperative examinations were confirmed by a radiologist and chief surgeon, who confirmed the number, size, and calcification of carcinoma and evaluated the lymph node state. Metastatic lymph nodes were suspected when lymph nodes showed increased size (>0.5 cm), demarcation of the cortex and medulla was unclear or the structure of the medulla disappeared, gravel-like calcification or cystic degeneration, rounded bulging shape, and abnormal blood flow or vascularity. Hashimoto’s thyroiditis (HT) was diagnosed when the thyroid gland was diffusely enlarged with hypoechogenicity, and the gland parenchyma was inhomogeneous with grid-like or compartment-like changes or appeared abnormal TPOAb, TGAb, TRAb results (8, 9). A gene mutation detection kit (Shanghai Anjia Biological Technology Co., Ltd.) was used to detect BRAF, TERT, HRAS, KRAS, NRAS mutations and RET/PTC rearrangements according to the manufacturer’s instructions. The scope of CLND was superior to the hyoid bone, lateral to the carotid sheath, inferior to the sternum notch or the innominate artery, medial to the trachea and dorsal to the prevertebral fascia.

### Statistical analysis

The t test (normally distributed data) and Mann−Whitney test (nonnormally distributed data) were used to compare continuous data, and the *χ* ^2^ test was used to compare categorical data. Multivariate analysis was carried out with logistic regression; therefore, independent predictors were used to construct a predictive model to evaluate the CLNM risk of cN0 PTMC.

Then, we developed a nomogram as a pictorial representation of the model. The nomogram featured a scoring range at the top from 0 to 100 for each factor variable. Predictor factors are shown below, and bars scaled their effects, displaying the importance of each factor clearly and enabling the awarding of points for each significant clinical feature. Each predictor point’s overall value and the corresponding probability of CLNM can be read from the bottom 2 rows.

The predicted accuracy and conformity of the model were assessed using the calibration curve and receiver operating characteristic (ROC) curve. The goodness of fit of the model was evaluated using the Hosmer−Lemeshow test. The net benefit for patients was shown by decision curve analysis (DCA), and 1000 bootstrap repetitions were performed in both discrimination and calibration. In the statistical analyses, P<0.05 was considered significant. Statistical analyses were performed using SPSS (version 22.0) and R (version 3.4.1).

## Results

### Baseline characteristics

**Figure 1:**
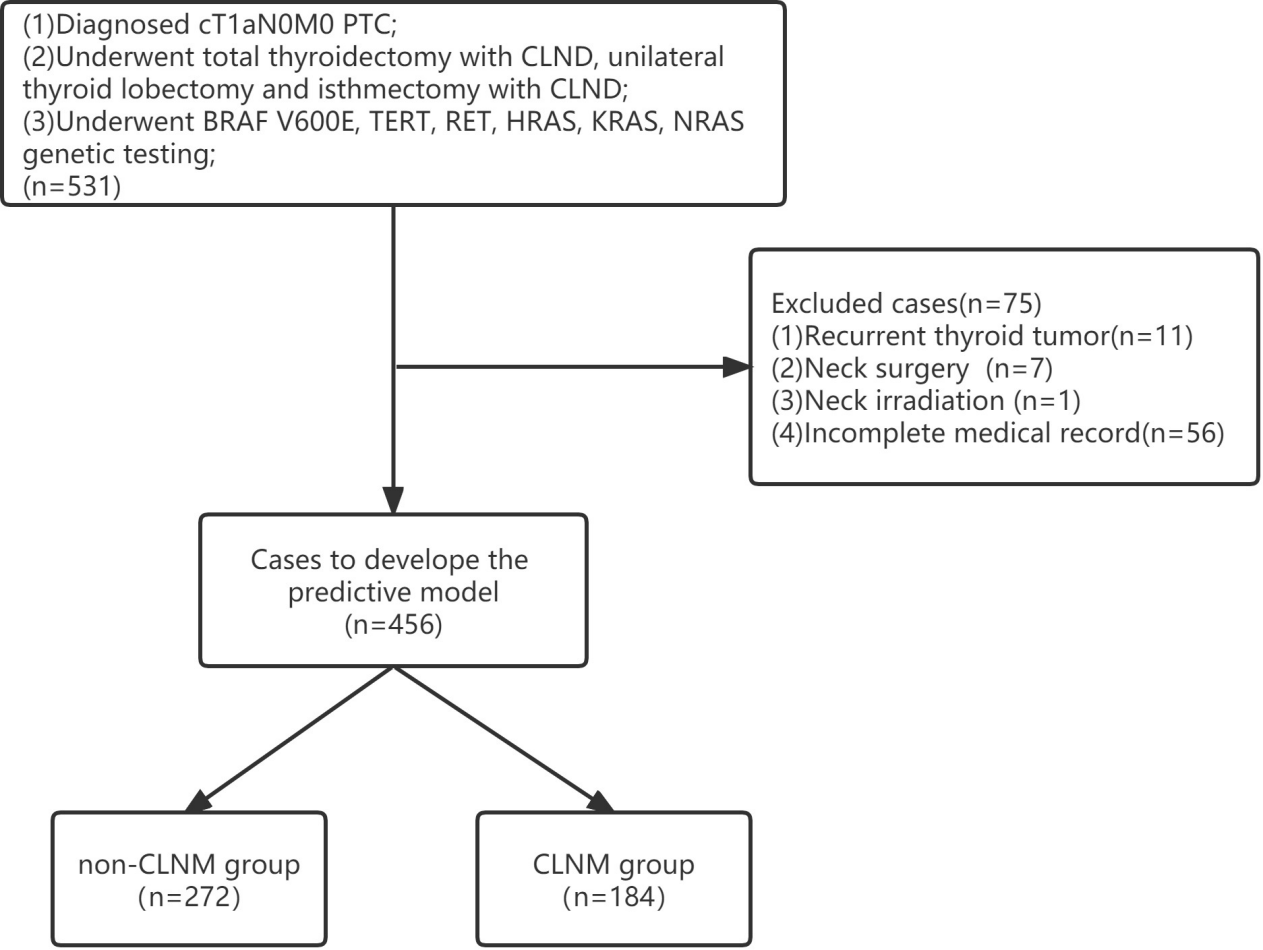
Flow chart for patient selection.

Clinical information from a total of 531 patients was acquired from the Affiliated Hospital of Qingdao University for the training cohort, and 75 met the exclusion criteria due to the absence of medical records and recurrent thyroid tumors (**Figure 1**). Therefore, 456 patients were enrolled in this cohort (97 males, 359 females; mean age 43.04 ± 13.68 years). Among them, 184 patients (40.35%) had CLNM. Clinical information from a total of 139 patients was acquired from the Affiliated Hospital of Jining Medical University for the validation cohort (21 males, 118 females; mean age 44.47 ± 11.54 years). Among them, 48 patients (34.53%) had CLNM. Finally, 595 patients were included in this study. The patients’ clinical, pathology and molecular characteristics are listed in **Table 1**.

**Table 1:** Baseline characteristics of all patients.

	Training cohort (n=456)			Validation cohort (n=139)		
	Non-CLNM group (n=272)	CLNM group (n=184)	P	Non-CLNM group (n=91)	CLNM group (n=48)	P
Age(Mean ± SD)(y)	42.39 ± 13.79	44.01 ± 13.50	0.216	46.40 ± 11.60	40.81 ± 10.58	0.008
Sexuality			0.006			0.176
Male	226	133		80	38	
Female	46	51		11	10	
Surgery Method			0.669			0.176
Unilateral	187	123		60	37	
Bilateral	85	61		31	11	
HT			0.006			0.054
Absence	177	142		51	26	
Presence	95	42		40	22	
Calcification foci			0.167			0.930
Absence	167	101		50	26	
Presence	105	83		41	22	
Tumor size (Mean ± SD)(mm)	5.66 ± 2.13	6.27 ± 2.36	0.004	6.98 ± 2.20	5.26 ± 2.52	0.000
Tumor number (Mean ± SD)	1.07 ± 0.29	1.07 ± 0.35	0.119	1.17 ± 0.48	1.10 ± 0.30	0.311
Extrathyroidal infiltration			0.001			0.002
Absence	224	126		82	33	
Presence	48	58		9	15	
BRAF V600E mutation			0.044			0.004
Negative	137	75		52	15	
Positive	135	109		39	33	
TERT promoter mutation			0.001			0.009
Negative	262	161		86	38	
Positive	10	23		5	10	
RET mutation			0.396			0.765
Negative	258	171		82	44	
Positive	14	13		9	4	
HRAS mutation			0.202			0.243
Negative	266	176		89	45	
Positive	6	8		2	3	
KRAS mutation			0.504			0.545
Negative	265	181		89	48	
Positive	7	3		2	0	
NRAS mutation			0.009			0.114
Negative	266	170		89	44	
Positive	6	14		2	4	

### Univariate and multivariate analysis

Within the training cohort, univariable analysis demonstrated that sex, HT, tumor size, tumor number, extrathyroidal extension, BRAF mutation, TERT promoter mutations and NRAS mutations were relative influencing factors of CLNM (P<0.05). These factors were included in multivariate logistic regression analysis. As shown in **Table 2**, the results revealed six independent factors, of which male sex, increased tumor size, extraglandular infiltration, TERT promoter mutations and NRAS mutation increased the risk of CLNM, while HT was a protective factor (P<0.05). Accordingly, a prediction model was built.

**Table 2:** Univariate and multivariate analysis of CLNM in the training cohort.

	Univariate Logistic Regression		Multivariate Logistic Regression	
	OR (95% CI)	P Value	OR (95% CI)	P Value
Age	1.009(0.995-1.023)	0.216	NA	NA
Sexuality	1.884(1.198-2.962)	0.006	4.360(2.446-7.769)	<0.001
Surgery method	1.091(0.731-1.628)	0.669	NA	NA
Calcification foci	1.307(0.894-1.910)	0.167	NA	NA
HT	0.551(0.360-0.843)	0.006	0.090(0.046-0.179)	<0.001
Tumor size	1.130(1.039-1.229)	0.004	1.208(1.093-1.334)	<0.001
Tumor number	1.597(0.887-2.877)	0.119	NA	NA
Extrathyroidal infiltration	2.148(1.383-3.337)	0.001	4.754(2.665-8.480)	<0.001
BRAF	1.475(1.011-2.153)	0.044	1.390(0.898-2.151)	0.140
TERT	3.743(1.737-8.067)	0.001	7.291(3.067-17.333)	<0.001
RET	1.401(0.643-3.054)	0.396	NA	NA
HRAS	2.015(0.687-5.907)	0.202	NA	NA
KRAS	0.627(0.160-2.459)	0.504	NA	NA
NRAS	3.651(1.376-9.685)	0.009	7.956(2.516-25.161)	<0.001

NA, Not Available.

### The nomogram for predicting PTC recurrence

**Figure 2:**
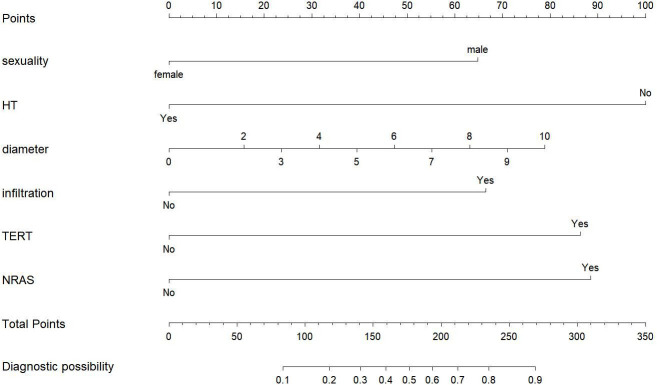
Nomogram for prediction model.

To visualize the model, we plotted a nomogram based on six independent factors (sex, HT, tumor size, extrathyroidal extension, TERT promoter mutations and NRAS mutation) in the training cohort. For each risk factor, an ascending line was drawn in the nomogram to obtain a point value in Line 1. The risk possibility of CLNM in cN0 PTMC patients might be estimated by plotting the total of all six points on the final risk axis (Line 8), as shown in **Figure 2**.

**Figure 3:**
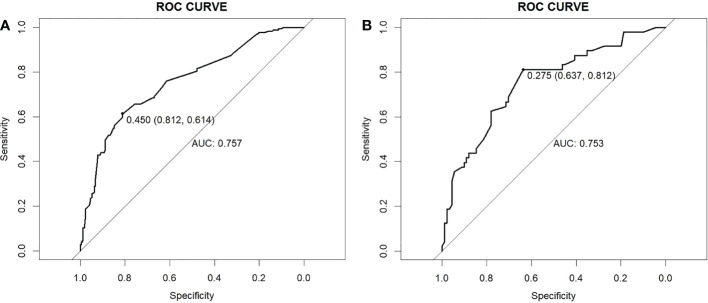
ROC curves. **(A)** Training cohort. **(B)** Validation cohort. ROC, receiver operating characteristic; AUC, area under the ROC curve.

Subsequently we drew the ROC curve to assess the discriminating ability of the model. The area under the ROC was 0.757 (95% CI, 0.711-0.802) and 0.753 (95% CI, 0.682-0.851) in the training cohort and validation cohort, respectively. The model demonstrated a strong capacity to discriminate CLNM in cN0 PTMC (this result is shown in **Figure 3**).

**Figure 4:**
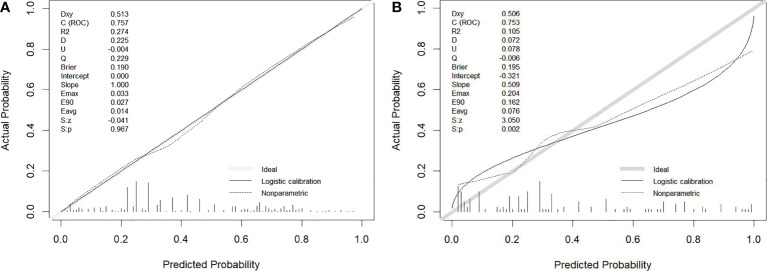
Calibration curve for prediction model. **(A)** Training cohort. **(B)** Validation cohort.

The calibration curve illustrated the model’s potent capacity to calibrate (**Figure 4**). In both the training cohort and the validation group, the model’s predicted values and the observed variables showed good agreement. According to the Hosmer−Lemeshow test, the model’s goodness of fit was 0.243.

**Figure 5:**
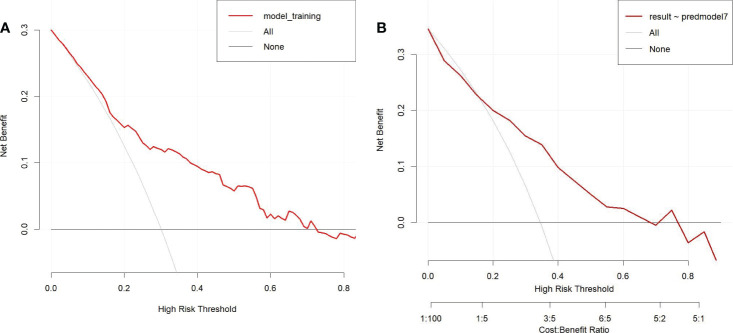
Decision curve analysis in prediction model **(A)** Training cohort. **(B)** Validation cohort.

To determine the net benefit of the nomogram, we built a decision curve analysis (DCA) (**Figure 5**). The curve demonstrated that the nomogram could be useful when the threshold probability of the patients was between 0.15 and 0.70. In the prediction model, the DCA’s net benefit was noticeably higher.

## Discussion

The most frequent pathological type of thyroid carcinoma is PTC (10), which is characterized by high differentiation and low malignancy. Most patients have a good prognosis, especially PTMC patients, whose cancer-related mortality rate is merely 0.34% after treatment (11, 12). Whereas PTMC patients have a high CLNM rate, studies have pointed out that 28% of PTMC patients are diagnosed with CLNM (13, 14). Thyroid cancer diagnosis and treatment guidelines (2022) published by the National Health Commission of the People’s Republic of China recommended prophylactic CLND should be performed for PTC patients as long as the parathyroid gland and recurrent laryngeal nerve can be protected (15). Moreover, the 2021 Chinese Society of Clinical Oncology (CSCO) Guidelines of Differentiated Thyroid Cancer recommended that PTMC patients with metastatic lymph nodes should undergo CLND (16). However, some studies still show that preventive dissection does not improve patient survival (17), and patients cannot benefit from it; conversely, it will increase the risk of nerve injury and hypoparathyroidism (18). How can metastasis be discriminated? Currently, the most common preoperative diagnostic method is ultrasound, the sensitivity of which for CLNM diagnosis is only 10.9%-38% (19). No practical way was found to raise the CLNM detection rate before surgery, which means that a large number of cN0 PTMC patients actually belong to pN1. Accordingly, identifying the risk factors for CLNM and evaluating the preoperative lymph node state accurately are of major clinical importance in cN0 PTMC patients, and some unnecessary prophylactic CLND can be avoided.

We conducted a large retrospective study including 595 patients from 2 independent medical centers and established a prediction model to assess the risk of CLNM in cN0 PTMC. The results showed that sex, HT, tumor size, extrathyroidal extension, TERT promoter mutations and NRAS mutation were independent factors.

Previous studies have shown that the incidence of PTC in males is lower than that in females, but the incidence of CLNM is higher (20). This is consistent with our findings that males showed a significant trend of CLNM (P < 0.001).

Tumor size reflects the stage and prognosis, such as lymph node status (5). In our study, the risk of CLNM in PTC increased with the enlargement of tumor size (P < 0.001), similar to other studies.

Extraglandular infiltration refers to the thyroid capsule being invaded by the tumor, and the tumor is more prone to infect lymph nodes through the thyroid capsule’s surface lymphatic channels after it has been breached (21). This result is consistent with our findings (P < 0.001).

HT is characterized by enrichment of inflammatory factors, also known as autoimmune thyroiditis. It produces a significant amount of lymphocytes and macrophages, both of which naturally possess antitumor activity, and the autoimmune antibodies released by HT-related lymphocytes can also destroy the follicles, contributing to their shrinkage and fibrosis (22, 23). Therefore, PTC tissues with HT usually exhibit a microscopic fibrous cell layer wrapping around the primary lesion, indicating that the combination of HL has a limiting effect on the progression of the primary lesion, which can significantly impede the spread and infiltration of tumor cells (24), reduce the invasiveness of the primary lesion (25), and decrease CLNM chance (26). Kim’s studies have shown that PTC patients with HT have lower clinical stage, lower incidence of vascular invasion, lower incidence of CLNM and better prognosis (27). Jara found that the incidence of CLNM in PTC patients with HT was 39% lower than that of PTC alone (28). Zhu analyzed 763 PTMC patients and showed that the presence of HT reduced the lymph node metastasis rate in PTMC patients (29). These results coincide with our study (P < 0.001). It is worth mentioning that the effect of HT may vary depending on the criteria used to define HT, Konturek found that the CLNM rate was four times higher in PTC patients coexisting with HT than non-HT patients (30).

The rapid development of fine-needle aspiration techniques makes preoperative genetic testing possible. Thus, surgeons can obtain preoperative genetic test results, such as BRAF gene mutation, TERT gene mutation, HRAS gene mutation, KRAS gene mutation, NRAS gene mutation and RET/PTC rearrangement.

BRAF, one of the three serine-threonine kinase RAF genes (the others being ARAF and CRAF), is crucial to the mitogen-activated protein kinase (MAPK) pathway (31). BRAF V600E is reported to be the most common mutation in PTC, with a probability of 29-69% (32). Although some studies revealed that BRAF genetic testing was valuable in PTC diagnosis (33), its utility in PTC prognosis remains controversial (34). According to several researchers, BRAF mutation is related to aggressiveness, such as lymph node metastases (35). In our study, the mutation incidence in the CLNM group (59.24%) was higher than that in the non-CLNM group (49.63%), but BRAF V600E mutation was not a relative influencing factor in CLNM (P=0.140). Probably because some BRAF V600E mutation testing was conducted preoperatively when patients could not be definitively diagnosed with PTC by US-FNAB pathology testing, which perhaps increased the proportion of BRAF V600E mutations patients inadvertently and affected its efficacy in CLNM diagnosis.

TERT promoter mutation has been deeply studied in various cancers, such as nervous system tumors, hepatocellular carcinoma, and bladder cancer (36). Recently, research on thyroid carcinoma has increased. Some articles have reported that the incidence of TERT promoter mutations is approximately 7.5-27% in PTC (37), and the mutation is correlated with particularly aggressive clinicopathological parameters, such as extrathyroidal extension, larger tumor size, and lymph node metastases (38–40). A meta-analysis study including 173 TERT promoter mutant and 1587 TERT promoter wild-type thyroid carcinoma patients showed that the LNM probability ratios were 53.18% and 37.30%, respectively, and a significant association existed between TERT promoter mutation and LNM (41). In Yang’s study, the TERT promoter mutation rate was 10.6% (1027/9653), which was significantly associated with PTC LNM (42). And Liu demonstrated that TERT promoter mutations occurred frequently in follicular derived PTC and associated with aggressive disease and poor outcome (43). These studies suggest that TERT promoter mutations can promote lymph node metastasis, which is consistent with our results. Nevertheless, most of the studies focus on advanced thyroid carcinoma (44), and studies on PTMC, especially CLNM, have rarely been conducted. In our study, TERT promoter mutation played an important role in diagnostic value and was an independent predictor of CLNM (p<0.001).

The second most frequent mutation is a RAS mutation in preoperative thyroid FNA. RAS mutations include 3 subtypes: HRAS, KRAS, and NRAS, the most common of which is NRAS (45). RAS mutations promote invasion mainly in follicular tumors, relate to local lymph node metastasis and distant metastasis (46). However, the encapsulated follicular type of papillary carcinoma, which is typically indolent and has a good prognosis, also frequently exhibits RAS mutations (47, 48). RAS mutations may only be present in thyroid carcinomas that are well-differentiated and prone to dedifferentiation and metastasis, but it is unlikely that this point mutation will serve as a general prognostic indicator for all varieties of thyroid carcinoma (49). In our study, NRAS was an independent factor for cN0 CLNM (p<0.001), perhaps just because of a specific subset, such as follicular papillary carcinoma.

There were many predictive models for assessing CLNM possibility in cN0 PTMC, but their models only incorporated clinical baseline data such as tumor characteristics, ultrasound and CT examination features or just included BRAF mutation result (50–52). Our study built the first predictive model that incorporates such molecular markers and included a large sample size. Finally, we identified 6 independent factors from preoperatively determinable clinical data and molecular markers and then built a predictive model to quantify the likelihood of CLNM in cN0 PTMC patients. The model exhibits good discrimination, calibration and clinical usefulness.

Nevertheless, it also had several limitations. First, our data were all obtained in one province, selection bias is inevitable, and the distribution of pathological subtypes and clinical features may differ in other regions. Second, the molecular pathology markers that were included in this study were limited, and important markers such as PTEN mutation, TRK rearrangement, and PAX8/PPARγ rearrangement were not involved. Third, our study requires further validation in prospective studies, and we are proceeding with a larger-scale, multicenter study in follow-up research. We hope our predictive model can provide valuable information for clinicians about facilitating the individualized prediction of CLNM in cN0 PTMC and guiding precise treatment.

## Data availability statement

The original contributions presented in the study are included in the article/supplementary material. Further inquiries can be directed to the corresponding author.

## Author contributions

TM designed the study, analyzed the data, and commented on the manuscript at all stages. LW and XZ collected the data. YS provided the research direction. All authors contributed to the article and approved the submitted version.
